# Unlocking the Functional Food Potential of *Capparis spinosa*: Optimizing Ultrasound Extraction, Phytochemical Analysis, and Assessing Antioxidative Activities

**DOI:** 10.3390/foods14101768

**Published:** 2025-05-16

**Authors:** Abdulhakim Naffati, Aleksandra Mišan, Saša Kenig, Katja Kramberger, Zala Jenko Pražnikar, Ana Petelin, Aleksandra Cvetanović Kljakić, Abdelmoumen Benmerzoug, Nasrien Elfarrah, Alena Stupar

**Affiliations:** 1 Libyan Medical Research Centre, Az Zawiyah 023-15583, Libya; abdul.naffati@gmail.com (A.N.); ali.nasrien@gmail.com (N.E.); 2 Institute of Food Technology, University of Novi Sad, Bulevar cara Lazara 1, 21000 Novi Sad, Serbia; aleksandra.misan@fins.uns.ac.rs; 3 Faculty of Health Sciences, University of Primorska, Polje 42, SI-6310 Izola, Slovenia; sasa.kenig@fvz.upr.si (S.K.); katja.kramberger@fvz.upr.si (K.K.); zala.praznikar@fvz.upr.si (Z.J.P.); ana.petelin@fvz.upr.si (A.P.); 4 Faculty of Technology, University of Novi Sad, Bulevar cara Lazara 1, 21000 Novi Sad, Serbia; a.c.istrazivac@gmail.com; 5 Department of Biological Sciences, Faculty of Nature and Life Sciences, University of Kasdi Merbah–Ouargla, Ouargla 30000, Algeria; benmerzoug.abdelmoumen@ens-ouargla.dz

**Keywords:** *Capparis spinosa*, ultrasound-assisted extraction, rutin content, antioxidative properties, reactive oxygen species, Caco-2 cells, Hep G2 cells

## Abstract

This study explores the potential of ultrasound-assisted extraction (UAE) for efficiently recovering bioactive compounds, particularly rutin, from the often-overlooked leaves of *Capparis spinosa*. By fine-tuning important extraction parameters such as ultrasound power, temperature, and ethanol concentration, this research achieved maximum yields of total phenolic content and rutin, establishing these leaves as a sustainable and valuable source of phytochemicals for applications in functional foods, nutraceuticals, and pharmaceuticals. High-performance liquid chromatography (HPLC) analysis revealed a high rutin content (15.51 mg/g DW), accompanied by considerable amounts of quercetin-3-glucoside and catechin. In vitro assays revealed the extract’s strong antioxidative properties, effectively safeguarding Caco-2 and Hep G2 cells against oxidative stress and significantly lowering reactive oxygen species levels. Additionally, the extract enhanced the expression of vital antioxidative enzymes and demonstrated no toxicity at the tested concentrations, confirming its safety for dietary supplement use. These findings underscore the extract’s strong safety profile and notable bioefficacy, reinforcing its suitability for incorporation into health-oriented formulations such as functional foods, dietary supplements, or nutraceutical products. Beyond demonstrating biological relevance, this research also advances the sustainable valorization of *Capparis spinosa* leaf biomass—an underutilized resource with high phytochemical potential—while contributing to the development of innovative, plant-based strategies for disease prevention and overall health enhancement.

## 1. Introduction

The rising interest in natural, plant-based compounds highlights a growing consumer understanding of the relationship between diet and health, resulting in driving demand for products that offer more than just basic sustenance. Consequently, the incorporation of herbal extracts and bioactive phytochemicals into food, nutraceutical, and pharmaceutical products has gained significant attention from both industry and academia. Incorporation of herbal-based ingredients into functional food formulations has become a strategic approach to harness the synergistic benefits of nutrition and targeted bioactive compounds.

The shift in dietary preferences has generated numerous research efforts focused on the therapeutic potential of various plant-derived compounds. Among the various herbal sources of bioactive compounds being investigated, *Capparis spinosa*, traditionally recognized for its medicinal and culinary value, and commonly known as the caper bush, has emerged as a promising candidate due to its rich phytochemical profile and documented health benefits. *Capparis spinosa* L. (*C. spinosa*) is one of the most popular edible herbs in *Capparidaceae* family [1]. The caper bush is a resilient, high-value crop renowned for its ability to thrive in harsh, arid environments and adapt to climate change challenges [2]. Caper bushes are found abundantly in the wild, particularly in the Mediterranean basin, as well as parts of Asia, Africa, and the Middle East, contributing to its long-standing culinary and traditional medicinal applications in these regions [3].

The relationship between the functional components present in the *Capparidaceae* family and their associated health benefits are fundamental to understanding their potential applications in nutrition and medicine. The diverse phytochemicals found in capers, such as flavonoids, phenolic acids, and glucosinolates, have demonstrated antioxidant, anti-inflammatory, antimicrobial, and neuroprotective properties [1,2,3]. In addition to its well-studied flower buds and fruits, the leaves represent an underutilized plant part with promising phytochemical richness. Phytochemical analyses have revealed that *C. spinosa* leaves are particularly abundant in polyphenolic compounds, especially flavonoids such as rutin, quercetin-3-glucoside, and catechin. These bioactive molecules are known for their diverse pharmacological activities, including potent antioxidant, anti-inflammatory, antimicrobial, and cytoprotective effects [4]. Studies indicate that caper and its bioactive compounds may help manage metabolic conditions like diabetes and hyperlipidemia, while also exhibiting anticancer, hepatoprotective, and cardioprotective effects. It is used to treat various human ailments including inflammation, gastro-intestinal problems, anemia, liver dysfunction, rheumatism, as well as being used as an antispasmodic analgesic, antihaemorrhoidal, aperient, diuretic, expectorant, etc. [5,6,7]. One of the dominant compounds in caper, the flavonoid rutin, has been shown to exhibit potent antioxidant activity, scavenging free radicals and mitigating oxidative stress, which are key contributors to cancer development, one of the most prevalent illnesses today. The diverse array of polyphenolic compounds acts synergistically to enhance antioxidant defenses and offer protective effects against various diseases. For example, the combined action of rutin, quercetin-3-glucoside, and catechin not only mitigates oxidative stress, but also promotes the activity of endogenous antioxidant enzymes like catalase and superoxide dismutase, which are crucial for maintaining cellular health [1,8]. This positions *C. spinosa* as a promising candidate for further research and development as a plant-based therapeutic or functional food ingredient.

The selection of *C. spinosa* leaves as the focus of this research is based on several important considerations. Although traditionally used in culinary and medicinal applications, the leaves have remained largely overlooked in scientific studies, particularly in comparison to the more commercially valuable buds and fruits. Unlike these plant parts, mature leaves are abundantly available throughout the growing season, providing a consistent, renewable, and underutilized biomass. Their valorization not only aligns with the principles of the circular bioeconomy, by reducing waste and maximizing resource efficiency, but also adds functional value to *C. spinosa* cultivation. Leaves are notably rich in bioactive compounds such as rutin and quercetin, which are well-documented for their strong antioxidant and anti-inflammatory effects. Such properties are particularly important in addressing oxidative stress and chronic inflammation, both of which are key contributors to the development of various non-communicable diseases [3,9]. By investigating this underexplored plant part, the present study contributes to both the sustainable exploitation of *C. spinosa* and the identification of novel sources of health-promoting phytochemicals.

Accordingly, considerable research efforts have been dedicated to optimizing the extraction of bioactive compounds from C. spinosa, with a particular focus on maximizing the recovery of high-value constituents such as rutin. Emerging extraction technologies, especially ultrasound-assisted extraction (UAE), have shown great potential in enhancing the efficiency of phenolic compound recovery, including rutin [2]. The cavitation effect generated by ultrasound waves plays a crucial role by disrupting plant cell walls and promoting enhanced mass transfer of intracellular components [10,11,12]. When these bubbles collapse, they produce localized high temperatures and pressures, which disrupt plant cell walls and facilitate the release of intracellular compounds. This mechanism not only enhances extraction efficiency, but also reduces the time required for effective extraction. However, excessive ultrasound intensity can lead to the generation of an overabundance of cavitation bubbles, resulting in the co-extraction of unwanted substances, reduced solubility of the target compound, and possible thermal degradation of rutin at elevated temperatures [13]. While concerns regarding the potential oxidation of sensitive polyphenols during ultrasound extraction exist, our study will implement stringent control over careful optimization of extraction parameters, particularly temperature and ultrasound power. These optimizations are designed to minimize oxidative degradation and preserve the integrity of the extracted bioactive compounds. By carefully balancing extraction efficiency with the stability of phytochemicals, UAE presents a promising avenue for maximizing the benefits of *C. spinosa* leaves while minimizing the loss of their valuable constituents.

Thus, the aim of this study was to develop a scalable, ultrasound-assisted green extraction protocol for the valorization of bioactive compounds—particularly rutin—from the underutilized leaves of *C. spinosa*. Key extraction parameters, including ultrasound power, temperature, and ethanol concentration, were optimized using the Box-Behnken Response Surface Methodology to maximize the yield of phenolic compounds, with a focus on rutin content and antioxidant activity. Furthermore, the biological efficacy of the extract obtained under optimal conditions was evaluated through cell culture experiments to assess its potential in mitigating oxidative stress, a key factor in the development of various non-communicable diseases.

## 2. Material and Methods

### 2.1. Chemicals

Folin-Ciocalteau reagent, 2,2-diphenyl-1-picrylhydrazyl radical (DPPH•), Trolox (6-hydroxy-2,5,7,8-tetramethylchroman-2-carboxylic acid), and gallic acid were sourced from Sigma-Aldrich GmbH (Taufkirchen, Germany). Ethanol (96% *v*/*v*) and methanol (95% *v*/*v*) were provided by Zorka Pharma (Šabac, Serbia). All other chemicals were of analytical grade, and water was purified using a Milli-Q system from Millipore (Bedford, MA, USA).

### 2.2. Plant Material

In this study, the material of investigation was caper leaves. Fresh, mature leaves of *C. spinosa* were collected from wild-growing plants in the Jabal al Gharbi region, near the town of Jadu in western Libya (31°56′59.99″ N, 12°00′60.00″ E). Sampling was performed from at least 10 healthy, non-flowering plants randomly distributed within a 1.5 km radius to ensure representativeness. The plant material was identified based on morphological features according to standard botanical keys, and authenticated by experts at the Libyan Medical Research Centre, Az Zawiyah, Libya. After collection, the leaves were washed with distilled water, air-dried in the shade at room temperature (~25 °C) for seven days, and ground into powder using a laboratory mill (IKA MF 10.1). The dried leaf powder exhibited a moisture content of 7.8 ± 0.4% and was sieved to obtain a uniform particle size of approximately 250–500 µm. The sieved powder was then stored hermetically in sealed plastic bags until used for further analysis or experimentation.

### 2.3. Extraction Protocol

#### 2.3.1. Ultrasound-Assisted Extraction (UAE)

In all experiments, 10.0 g of capers leaves was mixed with 100 mL of (30–70%) ethanol in a 250 mL flask. UAE was carried out in a sonication water bath (Transsonic TI-H 15, Elma Schmidbauer GmbH, Singen, Germany) operating at a fixed frequency of 45 kHz. The ultrasonic power was varied between 30 and 90% corresponding to 60 to 80 W/L, with extraction temperatures ranging from 50 °C to 80 °C. The selected ranges were defined based on preliminary single-variable screening experiments, as well as previous literature reports on ultrasonic-assisted extraction of polyphenols from *C. spinosa* and other similar medicinal plants [2,3,14]. All parameters, including ultrasonic power, temperature, and time, were controlled via the instrument panel. The flasks were consistently placed at the same distance from the transducer, with no additional agitation applied. Various extraction times were tested in the preliminary experiments, and based on the results, the duration was ultimately standardized to 60 min, aligning with the recommendation of Boudries et al. [2].

Following extraction, the samples were centrifuged at 4000 rpm for 10 min and filtered through a 0.45 μm PTFE membrane filter for further analysis, without additional purification steps, in line with the study’s aim to evaluate the bioactivity of the crude extract.

#### 2.3.2. Experimental Design

In this study, a Box-Behnken Design (BBD) was employed to optimize the extraction of bioactive compounds from *C. spinosa* using UAE. The experimental design consisted of 15 experiments with three variables at three levels (−1, 0, and 1), and three replicates at the central point. The independent variables—ultrasonic power (30–90%), extraction temperature (50–80 °C), and ethanol concentration (30–70%)—were investigated to determine optimal extraction conditions. The extraction time was fixed to simplify the process and focus on isolating the effects of the other three variables. Response Surface Methodology (RSM) was applied to analyze the effects of these variables on the extraction process, and the analysis was performed using Design-Expert v11 software (Stat-Ease Inc., Minneapolis, MN, USA). The independent variables were coded to range from −1 to 1 for the normalization.

This study aimed to optimize the yield of polyphenolic compounds and antioxidant activity. The second-order polynomial model was used to evaluate the influence of each variable on the responses (Equation (1)):

(1) $$Y=\beta_{0}+\sum_{i-1}^{k}\beta_{i}X_{i}+\sum_{i-1}^{k}\beta_{ii}X_{ii}^{2}+\sum_{i}^{k-1}\sum_{j}^{k}\beta_{ij}X_{i}$$

where Y is the response variable, *X_i_* and *Xj* are independent variables, and *β_i_*, *β_ii_*, and *β_ij_* are the regression coefficients for intercept, linear, and quadratic and interception terms, respectively. Regression coefficients were obtained to describe relationship between the responses and the independent variables.

### 2.4. Spectrophotometric Analysis

#### 2.4.1. Total Phenolic Content

The total phenolic content (TPC) was determined using the spectrophotometric method [15]. Absorbance was measured at 750 nm using a spectrophotometer (Specord M40, Carl Zeiss, Jena, Germany). Gallic acid was used as the standard to construct the calibration curve, and TPC was expressed as gallic acid equivalent (GAE) in mg GAE/g of *C. spinosa*.

#### 2.4.2. Total Flavonoids Content

Total flavonoid content (TFC) was determined using a colorimetric based method [16]. The extract was mixed with sodium nitrite, aluminum chloride, and sodium hydroxide, and absorbance was measured at 510 nm using a spectrophotometer. Catechin was used for the calibration curve, and TFC was expressed as catechin equivalent (CE) (mg CE/g of caper leaves).

#### 2.4.3. Antioxidant Capacity

The extracts’ free radical scavenging potential was determined using the DPPH assay. This method involves monitoring the reduction of the DPPH• radical, which changes from a deep purple color to yellow upon reaction with antioxidants. The antioxidant activity was quantified and expressed in terms of micromoles of Trolox equivalent per gram of dry weight (µmol TE/g DW).

### 2.5. Chromatographic Analysis

#### Phenolic Profile Determination by HPLC

Phenolic compounds in the obtained extracts were analyzed using HPLC (Agilent 1200 series) with an Eclipse XDB-C18 column (4.6 × 50 mm, 1.8 μm) and a diode array detector (DAD) [17,18]. Chromatographic separation used a linear gradient of methanol (solvent A) and 1% formic acid in water (solvent B) with a flow rate of 1 mL/min at 30 °C. Detection was performed at 280 nm, 330 nm, and 350 nm, with spectra recorded from 190 to 400 nm. The method was validated for linearity, LOD, LOQ, precision, and accuracy. Phenolic compounds were identified by comparing retention times and spectra with standards, and quantified using an external standard method. Compounds without available standards were expressed as equivalents of similar phenolics. The calibration concentration ranges for the dominant phenolic compounds are as follows: rutin (0.010–20.0 µg/mL), quercetin (0.050–20.0 µg/mL), and catechin (0.010–30.0 µg/mL) with R^2^ values > 0.998.

### 2.6. Cell Culture Experiments Evaluating Extracts of Caper Obtained Under Optimal Conditions

#### 2.6.1. Cell Culture Selection and Culture Conditions

Caco-2 human colorectal adenocarcinoma cells (ATCC^®^ HTB37™, Manassas, VA, USA) and Hep G2 human liver cancer cells (ATCC^®^ HB-8065™) were cultured in Dulbecco’s Modified Eagle’s Medium (DMEM) with high glucose, supplemented with 10% fetal bovine serum (FBS). The cultures were maintained at 37 °C in a humidified atmosphere containing 5% CO_2_. For all experiments, the extract was sterile filtered and diluted in cell culture media, with concentrations expressed as *v*/*v* percentages [19].

#### 2.6.2. Cytotoxicity

Cell viability following exposure to the extract was assessed using the PrestoBlue™ reagent (Invitrogen™, Carlsbad, CA, USA). Hep G2 cells (5000 per well) and Caco-2 cells (10,000 per well) were seeded in 96-well plates and allowed to adhere overnight. After 24 h of exposure to the designated extract concentrations, PrestoBlue™ was added to each well and incubated for 30 min. Fluorescence was measured at an excitation/emission wavelength of 535/595 nm using the Infinite F200 spectrophotometer (Tecan Group Ltd., Zürich, Switzerland). Each concentration was tested in five parallels, and the experiment was repeated three times.

#### 2.6.3. Protective Effect Against Induced Oxidative Stress

The protective effect of the extract against induced oxidative stress was assessed using the 2′,7′-Dichlorofluorescin Diacetate (DCF-DA) assay [20]. Caco-2 (10,000 cells per well) and Hep G2 (5000 cells per well) were seeded in black, clear-bottom 96-well plates and allowed to adhere for 24 h. Cells were treated with the extract for 24 h, then washed with phosphate-buffered saline (PBS), and exposed to DCFH-DA at a final concentration of 50 μM along with freshly prepared tert-butyl hydroperoxide (t-BOOH, 250 μM in PBS). After 30 min of incubation at room temperature, the increase in fluorescence was measured at an excitation/emission wavelength of 485/535 nm using the Infinite F200 spectrophotometer (Tecan Group Ltd., Zürich, Switzerland). The increase in fluorescence was expressed as relative fluorescence, with the untreated control sample set as 1.

#### 2.6.4. Expression of Genes Related to Oxidative Stress

Caco-2 and Hep G2 cells were exposed to the extract for 24 h. RNA was isolated using Qiazol Lysis reagent (Qiagen, Hilden, Germany) according to the manufacturer’s instructions. Reverse transcription of 1 µg of RNA was performed using the cDNA Archive kit (Applied Biosystems, Foster City, CA, USA). Quantitative RT-PCR was conducted on a QuantStudio^®^ 3 Real-Time PCR System (Thermo Fisher Scientific, Waltham, MA, USA) using SYBR Green master mix and 40 ng of cDNA template. The following primer sequences were selected from PrimerBank [21]: superoxide dismutase 1 (SOD1; 48762945c1), catalase (CAT; 260436906c3), and glutathione reductase (GR; 305410788c1). The 18S rRNA was used as an internal control. RT-PCR reaction conditions were set at 50 °C for 2 min, 95 °C for 10 min, followed by 40 cycles of 95 °C for 15 s and 60 °C for 1 min. The results were analyzed using the ΔCt algorithm and presented as fold-change relative to untreated cells.

### 2.7. Statistical Analysis

Analysis of variance (ANOVA) was employed to evaluate statistical significance at a confidence level of *p* ≤ 0.05. To determine the regression model’s goodness of fit, ANOVA was performed with a significance level of *p* < 0.05, and model adequacy was assessed using the coefficient of determination (R^2^), coefficient of variance (CV), and *p*-values for the model, along with lack-of-fit tests, using Design-Expert v11 software (Stat-Ease Inc., Minneapolis, MN, USA). All results are expressed as mean ± standard deviation, based on at least three independent experiments.

## 3. Results and Discussion

The bioactive compounds and chemical profiles of various parts of the *Capparis* genus—including fruits, flower buds, shoots, and roots—offer significant potential for exploration. However, innovative extraction methods, particularly for *C. spinosa* leaves, remain largely unexplored [2]. As demand for plant-based extracts grows, developing efficient extraction technologies that enhance the yield of bioactive compounds is crucial. Additionally, the rising interest in polyphenol-rich products for their health benefits highlights the need for better commercial extraction and analysis methods. Accordingly, this study discusses how to overcome this gap by optimizing the UAE technique to enhance the recovery of bioactive compounds from *C. spinosa* leaves.

### 3.1. Impact of Different Parameters on Extraction of Phenolics and Antioxidant Activity of the Extracts

The extraction of phenolic compounds and the assessment of antioxidant activity from the leaves of *C. spinosa* are influenced by various parameters. Table 1 presents the results obtained under various extraction conditions, including the phenolic content and antioxidant activity associated with each specific extraction condition.

As demonstrated by Rani et al. [22], the efficiency of phenolic compound extraction is significantly influenced by the polarity of the solvent used. Polar solvents such as ethanol and methanol have shown superior performance in extracting phenolic and flavonoid compounds, primarily due to their high solubility for polar bioactives. Among them, aqueous ethanol was selected as the optimal solvent for further optimization, offering an ideal compromise between extraction efficiency and safety. Building upon these findings, ethanol concentration was selected as one of the key variables for further extraction optimization.

The effect of ethanol concentration on polyphenol extraction showed some interesting results. Using a lower ethanol concentration (30%) with higher ultrasound power (90%) gave the highest TPC and antioxidant activity. For example, at 50 °C with 30% ethanol and 90% ultrasound power, the TPC reached 23.14 mg GAE/g, and antioxidant activity was 20.61 mg TE/g in the DPPH test. This suggests that phenolic compounds play a key role in the antioxidant capacity of the extracts. On the other hand, higher ethanol concentrations (like 70%) resulted in lower yields of phenolic compounds and flavonoids, probably because more polar compounds were less soluble and harder to extract. For example, at 80 °C with 70% ethanol, the TPC dropped to 18.75 mg GAE/g. With 70% ethanol, 80 °C, and 30% ultrasound power, high total flavonoid content (101.38 mg GAE/g) and rutin content (14.74 mg/g) were seen. However, very high or low ethanol concentrations did not significantly improve TPC or antioxidant activity, suggesting that a balance in solvent polarity is important for the best polyphenol extraction. Moderate ethanol concentrations (50–70%) were better for getting higher yields of TPC and rutin. The highest rutin content (14.78 mg/g) was achieved at 80 °C with 50% ethanol and 60% ultrasound power. DPPH scavenging activity remained fairly consistent across conditions (around 20–21.8 mg TE/g), showing that antioxidant properties remained strong throughout. The highest DPPH activity (21.87 mg TE/g) occurred at 65 °C, 50% ethanol, and 60% ultrasound power, which also matched the highest TPC value, indicating a strong link between extracted polyphenols and antioxidant activity.

Higher extraction temperatures, especially around 80 °C, generally led to higher TPC yields, with the highest TPC (23.14 mg GAE/g) found at 80 °C, 30% ethanol, and 30% ultrasound power. This could be due to better solvent diffusion and cell wall breakdown at higher temperatures, which helps release more phenolics. The results also showed that moderate temperatures (50 °C to 65 °C) tended to boost both TPC and antioxidant activity. For example, at 65 °C with 50% ethanol, the TPC was 22.25 mg GAE/g, indicating that moderate temperatures helped dissolve phenolic compounds without breaking them down, unlike at higher temperatures (80 °C), where TPC dropped to 20.08 mg GAE/g. However, the total flavonoid content (TFC) did not follow the same pattern. At higher temperatures (80 °C), TFC values decreased. Moderate temperatures, especially between 50 °C and 65 °C, resulted in higher TFC values. The highest TFC (132.14 mg GAE/g) was seen at 65 °C, suggesting that certain polyphenolic compounds may be better extracted at lower thermal conditions, possibly because high temperatures can cause thermal degradation or transformation of heat-sensitive compounds. For example, at 50 °C with 30% ethanol, the TFC was highest, indicating that this temperature range is ideal for extracting flavonoids without degrading them.

Similarly, Kruszewski and Boselli [23] concluded that the impact of temperature on extraction efficiency is closely linked to the solvent system and the thermal stability of the target compounds. In their experiments, for the water–ethanol solvent, the best results were achieved at 35 °C, a lower temperature than the one obtained to be optimal in our experiments. However, it is important to note that their focus was on anthocyanins, a class of compounds considerably more sensitive to heat compared to rutin and other phenolics targeted in our study.

Ultrasound power plays a vital role in enhancing extraction efficiency by breaking down plant cell walls and improving solvent penetration. As shown in Table 1, increasing ultrasound power leads to higher yields of phenolics and flavonoids. Increasing ultrasound power from 30% to 90% significantly enhances both TPC and TFC. The highest values for both TPC (23.1436 mg GAE/g) and TFC were achieved at 50 °C, 30% ethanol, and 90% ultrasound power. Conversely, lower ultrasound power (60%) produced significantly lower phenolic and flavonoid yields. For example, at 80 °C with 30% ethanol, TPC decreased to 20.08 mg GAE/g. The data also highlight that ultrasound power, especially at moderate temperatures, significantly increases rutin yield. At 50 °C with 30% ethanol, rutin concentration was 10.034 mg/g with 30% ultrasound power, but increased to 14.15 mg/g when ultrasound power was raised to 90%, suggesting that ultrasound-assisted extraction is an effective way of maximizing the extraction efficiency of rutin.

While many studies report a decrease in extraction efficiency at higher ultrasound power levels, this trend was not observed in our case. Interestingly, similar findings were reported by Hiranpradith et al. [11], who suggested that under certain experimental conditions, the energy delivered may be insufficient to significantly enhance the extraction process. This was attributed to the use of an ultrasonic bath, which generally transmits less energy to the sample compared to probe-based systems, due to energy dissipation within the medium.

### 3.2. Parameters Optimization

The experimental data presented in Table 1, coupled with the regression analysis (Table 2), provides a clear understanding of the relationships between these factors and how they influence extraction efficiency. The regression analysis of bioactive compound extraction from *C. spinosa* using UAE revealed several significant linear, interaction, and quadratic effects. The response variables analyzed include total phenolic (TP), total flavonoid (TF), antioxidant activity (DPPH test), and rutin content. Regression coefficients and model diagnostics such as the coefficient of determination (R^2^) and coefficient of variance (CV) are presented in Table 2, illustrating how extraction conditions impact the yield and quality of these compounds.

For total phenolic content, the model was found to be significant, with temperature (β_1_) and ethanol concentration (β_2_) identified as the primary drivers (*p* = 0.0249 and *p* = 0.0078, respectively). The observed significant interaction between temperature and ethanol concentration (β_12_, *p* = 0.0225) indicates that these two factors work together to enhance phenolic extraction. In contrast, ultrasound power (β_3_) showed a borderline effect (*p* = 0.0875), and the lack of significant quadratic terms implies that the relationships in this case are mostly linear.

The analysis of total flavonoid content exhibited some differences. While the overall model was significant (*p* = 0.0060), ethanol concentration emerged as the most influential factor (*p* = 0.0003). The significant quadratic term for ethanol concentration underscores a nonlinear relationship, indicating that precise control is required for optimal extraction. In this case, neither temperature nor ultrasound power displayed significant main effects (*p* = 0.1312 and *p* = 0.4998, respectively), and there were no significant interaction terms. Although the quadratic term for temperature approached significance (*p* = 0.0574), the data suggest that ethanol concentration is the key parameter for flavonoid extraction, emphasizing the need for careful management.

Regarding antioxidant activity assessed by DPPH, although the linear effect of ultrasound power (β_3_ = 0.26) was not statistically significant, its interactions with other variables were crucial for enhancing antioxidant activity. The positive interaction term (β_23_ = 0.44, *p* < 0.05) suggests that specific combinations of extraction parameters can lead to synergistic effects that increase the antioxidant capacity of the extracts. This indicates that the overall extraction process benefits from the interactions among temperature, ethanol concentration, and ultrasound power, even if individual parameters show limited effects.

In terms of rutin content, the model demonstrated robust significance (*p* = 0.0124), with temperature and ethanol concentration again playing dominant roles (*p* = 0.0010 and *p* = 0.0032, respectively). Unlike TPC, ultrasound power did not have a significant impact on rutin extraction (*p* = 0.5929), and the model revealed a linear relationship for rutin recovery, indicating that fine-tuning temperature and ethanol concentration is essential for maximizing the extraction of specific phenolic compounds.

The linear, interaction, and quadratic effects identified in this study provide valuable insights into how various variables affect the extraction process. The reliability of the models is supported by high R^2^ values and low CVs, making them effective tools for predicting extraction outcomes and optimizing protocols for better yields. Across all responses, total phenolic content, rutin, and total flavonoids, the regression models demonstrated statistical significance, with *p*-values ranging from less than 0.0001 to 0.0319. The adjusted R^2^ values, which range from 0.69 to 0.98, indicate a strong fit and explain a substantial portion of the variance in the results. This strong correlation is further validated by relatively low CVs for most responses, suggesting precise model predictions. Although some lack-of-fit results, such as the *p*-value of 0.0618 for flavonoids, suggest minor areas for improvement, the overall non-significant lack-of-fit tests indicate that the models remain reliable for making predictions within the tested conditions. This underscores the effectiveness of the regression models in guiding the extraction process and optimizing results.

Taken together, that ethanol concentration showed to be a critical parameter across all responses, especially for flavonoid extraction, with only marginal contributions from temperature and ultrasound power. For TPC and rutin, optimizing temperature in tandem with ethanol concentration appears essential due to their strong interaction, while ultrasound power has lower influence.

Based on the experimental results and statistical analysis, numerical optimizations have been conducted in order to establish the optimum level of independent variables with desirable response of goals. For all responses, one optimal condition was obtained: 80 °C temperature, 62.23% ethanol, and 56.05% of ultrasound power. Determination of optimal conditions and predicted values was based on desirability function, D = 0.778, in order to focus on high rutin content. In order to verify the predictive mathematical model of the investigated process, experimental confirmation of the obtained results was performed on estimated optimal conditions. The predicted and observed values are presented in Table 3. The predicted results matched well with the experimental results obtained at optimal extraction conditions, which were validated by the RSM model with good correlation.

Similar findings were reported by Boudries et al. [2], who demonstrated that both experimental and modeled data confirmed a significant enhancement in extraction efficiency when ethanol was incorporated into the solvent system. Although temperature also had a positive influence, its effect was comparatively less pronounced in the extraction of phenolic compounds from *C. spinosa* flower buds.

To date, the content of total polyphenols, flavonoids, and flavonols has been extensively investigated in various edible aerial parts of the caper plant. According to Wojdyło et al. (2019) [5], flower buds exhibited notably high levels of total polyphenols (849.4 mg GAE/100 g FW), flavonoids (729.5 mg RE/100 g FW), and flavonols (691.6 mg RE/100 g FW), surpassing the concentrations observed in young caper shoots. In contrast, caper fruits presented considerably lower levels of these compounds, with total polyphenols at 119.2 mg GAE/100 g FW, flavonoids at 81.6 mg RE/100 g FW, and flavonols at 39.9 mg RE/100 g FW [5].

Although research on caper shoots and leaves are limited, some studies have explored their polyphenol content. Gull et al. (2019) [24] reported a relatively low total polyphenol concentration of approximately 28.7 mg GAE/100 g DW in shoots. Tlili et al. (2017) [25], in a study on methanolic extracts from *C. spinosa* leaves, reported total phenolics, flavonoids, and condensed tannins of 23.37 mg GAE/g DW, 9.05 mg QE/g DW, and 9.35 mg TAE/g DW, respectively. Further insights were provided by Fattahi and Rahimi (2016) [26], who applied response surface methodology to optimize extraction from *C. spinosa* leaves. The optimal conditions—49% ethanol, 51.8 °C, and a solvent-to-material ratio of 50 (*v*/*w*)—resulted in total polyphenol content of 27.44 mg GAE/g DW, total flavonoid content of 26.07 mg QE/g DW, and DPPH radical scavenging activity of 85.74%. In comparison, the present study optimized ultrasound-assisted extraction conditions at 80 °C, 62.23% ethanol, and 56% ultrasound power. Under these parameters, the obtained values were: total polyphenol content of 19.82 mg GAE/g DW, total flavonoid content of 99.26 mg RE/g DW, antioxidant activity of 21.54 mg TE/g DW (DPPH assay), and rutin content of 15.79 mg/g DW. While the TPC obtained was slightly lower than some values reported in the literature, the high flavonoid content and particularly high rutin concentration underscore the efficiency of the extraction method. Moreover, the specific quantification of rutin (15.79 mg/g DW) highlights the successful enrichment of this pharmacologically significant compound, which is not always individually assessed in earlier studies. It is important to note that TFC (as RE) encompasses multiple flavonoid constituents beyond rutin, reflecting the cumulative content of flavonoids in the extract. The optimized conditions employed in this research demonstrate a highly effective and sustainable approach to maximizing the recovery of phenolics and flavonoids from *C. spinosa*.

Differences between the present findings and literature values can be attributed to several factors, including the plant part analyzed, geographical origin, genotype, harvesting stage, and extraction methodology. Additionally, the biosynthesis of phenolic compounds and flavonoids is known to be influenced by abiotic stresses, such as heat, which further contributes to variability in phytochemical content [27].

### 3.3. HPLC Analysis

HPLC analysis was conducted on all extracts in order to determine how extraction conditions affect the polyphenolic profile. Under all experimental conditions, rutin was a dominant compound; therefore, it was included as a main compound in the optimization process.

**Figure 1:**
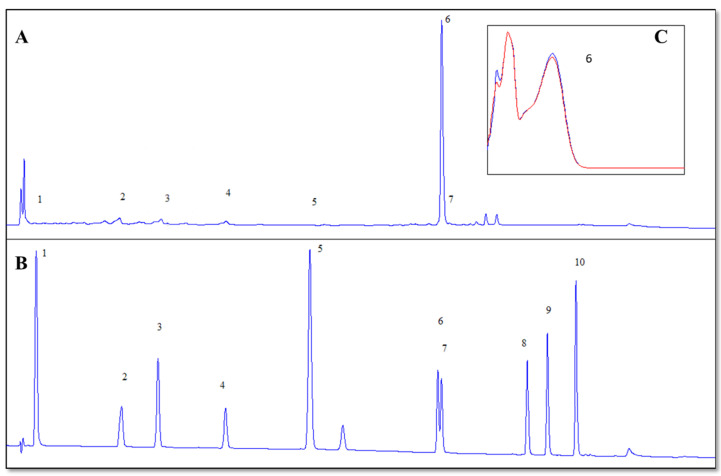
HPLC chromatogram of *C. spinosa* leaf extract obtained under optimal conditions: (**A**) sample extract, (**B**) standard mixture, and (**C**) overlaid UV spectra of rutin in the sample (blue) and standard (red). 1—Gallic acid, 2a—Catechin, 2b—Catechin equivalent, 3—Caffeic acid, 4—Syringic acid, 5—Sinapic acid, 6—Rutin, 7—Quercetin-3-O-glucoside, 8—Myricetin, 9—Luteolin, 10—Apigenin.

To ensure reliable compound identification, a mixture of authenticated analytical standards (rutin, catechin, caffeic acid, syringic acid, gallic acid, sinapic acid, and quercetin-3-glucoside) was used. Identification was based on matching retention times and UV–Vis absorption spectra of peaks in the sample extract to those in the standard mixture. Figure 1 presents the chromatogram of the extract obtained under optimal conditions (a), the standard mixture chromatogram (b), and the overlaid UV spectra of rutin from both standard and sample (c), confirming the identity of the main compound.

The HPLC analysis of the optimal extract revealed several key compounds, including catechin equivalents, caffeine, syringic acid, sinapic acid, rutin, and quercetin-3-glucoside, which are known for their potential health benefits (Table 4).

The phytochemical composition of *C. spinosa* has been extensively investigated, with several studies highlighting its richness in phenolic and flavonoid compounds. Among these, rutin consistently emerges as the dominant flavonoid. The antioxidant capacity and phenolic content of buds were found to be markedly higher than berries. In *C. spinosa* flower bud extract, rutin was the major flavonoid, followed by quercetine rhamnoside and kaempferol rhamnoside [26]. Gull et al. (2019) [24] and Tlili et al. (2017) [25] emphasized *C. spinosa* as a notable source of rutin and quercetin derivatives, with high pharmacological and nutritional potential. In the present study, HPLC analysis revealed a rutin content of 15.51 mg/g DW, emphasizing the effectiveness of the optimized ultrasound-assisted extraction protocol. This value is notably higher than the 3.96 mg/g DW reported by Tlili et al. (2017) [25], suggesting considerable variability that may be attributed to differences in extraction methods, solvent systems, plant material, and environmental or cultivation conditions. Although ultrasound power did not have a significant impact on rutin extraction (*p* = 0.5929), the higher rutin yield observed in this study highlights the potential advantages of ultrasound-assisted extraction in enhancing the recovery of specific bioactive compounds.

In addition to rutin, quercetin-3-glucoside (4.27 mg/g DW) was identified in the extract. This specific compound was not reported by Tlili et al. (2017) [25], although their analysis revealed other flavonols such as kaempferol (0.40 mg/g DW) and luteolin (0.78 mg/g DW). While quercetin derivatives are evident in both studies, differences in the types and concentrations of flavonoids underscore the influence of extraction techniques and the specific plant material analyzed. Furthermore, catechin was detected at 2.07 mg/g DW in the present study, which is notably higher than the 0.56 mg/g DW reported by Tlili et al. (2017) [25]. In contrast, gallic acid content in this extract was 0.04 mg/g DW, which is lower than the 0.14 mg/g DW observed by Tlili et al. Syringic acid (0.19 mg/g DW) and caffeic acid (0.25 mg/g DW) were also identified in the present extract. In contrast, Tlili et al. (2017) [25] did not report caffeic acid in their analysis and instead identified vanillic acid (0.27 mg/g DW) among the minor phenolic acids. These differences in phenolic acid profiles may reflect not only methodological variations such as the use of ultrasound-assisted extraction with ethanol versus maceration with methanol, but also differences in phenological stage of leaf collection, plant genotype, or eco-geographical growing conditions. A notable contrast is the presence of resveratrol, which was the second-most abundant compound (2.35 mg/g DW) in the extract analyzed by Tlili et al., yet it was not detected in the current study. Similarly, epicatechin (1.28 mg/g DW) and coumarin (1.33 mg/g DW) were reported by Tlili et al., but were not observed in the present analysis. This could suggest a selectivity of ultrasound-assisted extraction toward specific phenolic compounds, or it may indicate differences in compound solubility and extraction efficiency related to solvent polarity, matrix structure, or compound stability under ultrasonic conditions.

These variations may be attributed to differences in extraction methods (e.g., ultrasound-assisted extraction using ethanol vs. conventional maceration with methanol), solvent polarity, temperature, or ultrasound power intensity, all of which significantly affect the recovery and profile of phenolic compounds. Moreover, the high flavonol content observed in this study may be linked to the plant’s physiological responses to biotic and abiotic stressors. As noted by Stefanucci et al. (2018) [27], the accumulation of flavonols such as rutin, quercetin, and kaempferol often reflects the plant’s adaptive response to environmental pressures, including UV radiation, drought, temperature extremes, or pathogen attack. In addition, Kianersi et al. (2020) [28] demonstrated that exogenous application of salicylic acid and methyl jasmonate can significantly stimulate rutin biosynthesis in *C. spinosa* in a growth stage-dependent manner [28]. These findings suggest that targeted agronomic interventions, in combination with optimized extraction protocols, could further enhance the yield of valuable bioactive compounds in *C. spinosa*.

### 3.4. Cell Culture Response to Extract Treatment

**Figure 2:**
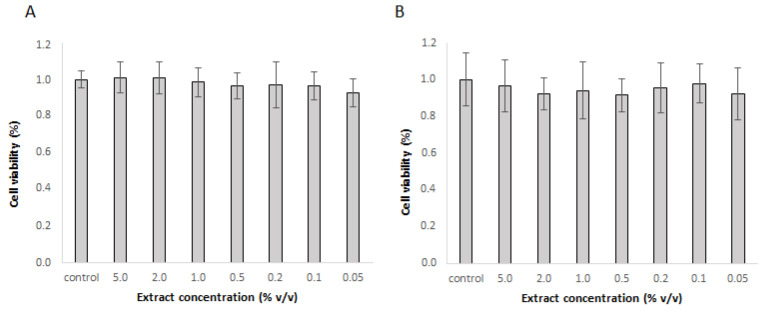
Cytotoxicity of different extract concentrations as determined by the PrestoBlue Assay in (**A**) Caco-2 cells and (**B**) Hep G2 cells. The mean ± SD of three separate experiments is presented.

Based on the results in Table 1, the extract prepared at optimal conditions, with the highest rutin level, was selected to test its properties in cell lines. First, cytotoxicity was tested in Caco-2 and Hep G2 cells. The extract was not toxic in concentrations up to 5% *v*/*v* in either cell line (Figure 2A,B). As none of the concentrations were toxic, the extract was used in 1% and 5% concentrations for further experiments.

**Figure 3:**
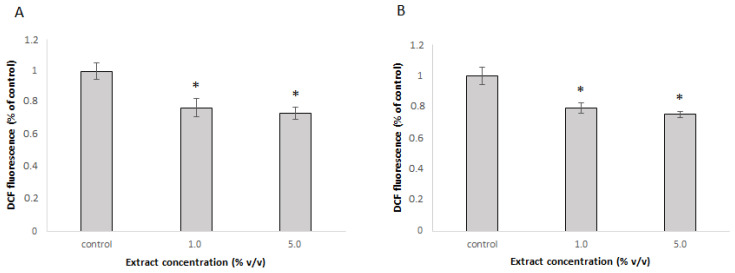
Protective effect of the extract against the oxidative stress induced with t-BOOH and measured with DCFH-DA assay in (**A**) Caco-2 cells and (**B**) Hep G2 cells. The mean ± SD of two separate experiments is presented, * *p* < 0.05.

In vitro results showed promising results for the antioxidative potential of the extract. The extract was further tested for its ability to protect cells against t-BOOH-induced oxidative stress. The DCFH-DA assay, which is based on the use of a fluorescent cell-permeable probe that reacts with reactive oxygen species (ROS) to form green fluorescent product, was used. Given that antioxidative effects in cell cultures depend on the ability of individual compounds to cross cell membranes and the presence of metabolic enzymes that may convert them, the protective effect of the extract against induced oxidative stress was evaluated in Caco-2 and Hep G2 cells. Caco-2 cells, which represent colon cells, were selected, as they are the first to encounter ingested substances. Hep G2 cells, representing liver cells, were chosen for their role in metabolizing bioactive compounds. These two cell types are therefore suitable for assessing the safety and potential beneficial effects of dietary substances. In Caco-2 cells, the extract had a significant protective effect in both tested concentrations and reduced the amount of ROS to 79% and 75% in 1.0% and 5.0% concentrations, respectively (Figure 3A). The results were similar in Hep G2 cells, where the amount of ROS was reduced to 77% with 1.0% extract and to 73% with 5% extract (Figure 3B).

The protective effect may be exerted through various mechanisms, including the direct action of bioactive compounds, as measured by in vitro tests, or the increased production of endogenous antioxidants. The protective effect of rutin as a dominant compound in optimized extract of *C. spinosa* and other compounds found in the extract may be mediated through various mechanisms, including the direct action of bioactive compounds, as demonstrated by in vitro tests, or through enhanced production of endogenous antioxidants. Rutin has been shown to modulate several cell signaling pathways involved in cell cycle regulation, apoptosis, and angiogenesis, effectively suppressing key features of cancer progression [29,30,31].

**Figure 4:**
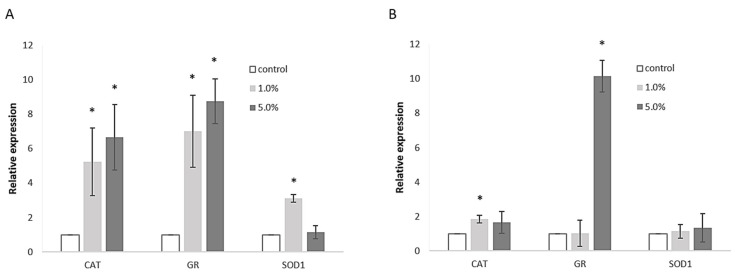
Expression levels of catalase (CAT), glutathione reductase (GR), and superoxide dismutase 1 (SOD1) in (**A**) Caco-2 cells and (**B**) Hep G2 cells. Expressions were measured by RT-PCR, normalized to 18S rRNA, and set as 1 for the control sample. Average ± SD is presented. * *p* < 0.05.

To further investigate these effects, RT-PCR was employed to assess the expression levels of catalase (CAT), glutathione reductase (GR), and superoxide dismutase 1 (SOD-1). In Caco-2 cells (Figure 4A), CAT expression was significantly elevated, showing a 5.3-fold increase with 1.0% extract and a 6.7-fold increase with 5.0% extract compared to control cells. GR expression was also notably up-regulated, with a 7.0-fold increase for the 1.0% extract and an 8.8-fold increase for the 5.0% extract. The effect on SOD-1 was less pronounced, with only a 3.1-fold increase observed at the lower extract concentration. In Hep G2 cells (Figure 4B), GR expression was markedly increased, showing a 10.2-fold up-regulation in response to the 5% extract, while the lower concentration resulted in a moderate 1.8-fold up-regulation of CAT. Other gene expressions remained unaffected.

The non-toxic nature of the extract at the tested concentrations suggests its safe incorporation into functional food products or use as a dietary supplement. Notably, the extract effectively protected cells from induced oxidative stress, as evidenced by significantly lower levels of reactive oxygen species in both cell types when pretreated with the extract. Since the extract was removed prior to the addition of t-BOOH in this experiment, the protective effect cannot be attributed to direct reactions between the bioactive compounds of the extract and t-BOOH in the cell culture media. However, the components absorbed into the cells may act through such mechanisms. Moreover, results from the RT-PCR experiments indicate that the extract not only provides direct protection, but also induces the expression of cellular antioxidative enzymes. This effect was more pronounced in Caco-2 cells, where all three tested gene expressions were significantly up-regulated. Less substantial effects in liver cells may be due to their already strong intrinsic antioxidant system and detoxification mechanisms, which may neutralize the active components. This includes phase I and phase II enzymes, known to be expressed in Hep G2 cells [32].

The observed protective effect and the induction of antioxidative enzymes can be attributed to the chemical components detected in the extract. Rutin, the main component, was consistently shown to protect cells from oxidative damage [33,34], partly by activating CAT and SOD [35]. Similarly, quercetin demonstrated efficient antioxidant capacity [36]. Quercetin increased the expression of CAT and decreased the expression of SOD at low concentrations, but this effect was reversed at high concentrations [35]. Such a phenomenon is most commonly observed for complex mixtures of bioactive compounds. According to the principle of hormesis, low concentrations of certain compounds stimulate adaptive cellular responses, but when higher concentrations are used, such compensation is no longer possible [37]. Therefore, the observed induced expression of SOD1 in Caco-2 cells and CAT in Hep G2 cells only at lower extract concentrations is not surprising.

## 4. Conclusions

This study underscores the remarkable potential of *C. spinosa* leaves as a sustainable and valuable source of bioactive compounds, particularly rutin, which was identified at a notably high concentration (15.51 mg/g DW) using an optimized ultrasound-assisted extraction protocol. Fine-tuning extraction parameters such as ultrasound power, temperature, and ethanol concentration proved effective in maximizing the recovery of phenolics, positioning these often-discarded leaves as a promising raw material for the development of functional foods, nutraceuticals, and pharmaceutical applications. Furthermore, the extract demonstrated potent antioxidant activity, as evidenced by substantial reductions in reactive oxygen species in both Caco-2 and Hep G2 cells under t-BOOH-induced oxidative stress. The protective effects are attributable not only to direct antioxidant mechanisms, but also to the upregulation of key endogenous defense enzymes, including catalase (CAT), glutathione reductase, and superoxide dismutase, particularly in intestinal cells. These findings underscore the extract’s potential to modulate oxidative stress-related pathways and enhance cellular resilience. Importantly, the extract exhibited no cytotoxicity at the tested concentrations, reinforcing its safety profile for dietary or therapeutic use. This combination of significant bioactivity, sustainable biomass sourcing, and scalable extraction methods presents a compelling case for the valorization of *C. spinosa* leaves as an underutilized agricultural byproduct with notable health benefits and economic value. The results provide a strong scientific foundation for future in vivo studies and the innovation of plant-based health products while highlighting the utility of green extraction technologies as effective tools for unlocking the therapeutic potential of botanical resources. The bioactive compounds recovered from *C. spinosa* leaves can be incorporated into functional foods, nutraceuticals, and pharmaceutical products, providing new opportunities for value-added utilization of this versatile plant.

## Figures and Tables

**Table 1:** Central composite design of the three-levels and three-variables and experimentally observed values of target responses.

Extraction Temperature (°C)	Ethanol Concentration (%)	Ultrasound Power (%)	TPC (mg GAE/g DW)	TFC (mg GAE/g DW)	DPPH (mg TE/g DW)	Rutin (mg/g DW)
80	30	30	23.1436	90.3909	20.6089	14.14833
65	50	60	22.2452	91.1791	21.1616	13.49059
80	30	90	20.3945	94.4752	21.1772	14.1438
80	50	60	20.0845	94.4752	21.3739	14.77886
65	50	60	19.4152	88.4563	21.1179	12.94625
50	50	60	19.1367	90.4482	21.3614	12.85552
65	70	60	18.8492	97.0834	21.3552	14.31617
65	50	30	18.7774	84.0997	21.0679	14.06215
80	70	30	18.7459	101.3826	21.3115	14.73804
65	30	60	18.7414	90.1759	21.2022	13.70832
50	30	30	18.7010	91.6520	21.0342	10.03402
65	50	60	18.6516	132.1364	21.8735	12.82831
50	30	90	18.6291	89.0152	20.9368	10.2064
50	70	90	18.2518	101.9988	21.4582	14.28895
65	50	90	17.9059	90.8638	21.3396	13.22295
50	70	30	17.6993	99.6486	20.0906	13.40894
80	70	90	17.0210	107.6595	21.3923	15.85394

TPC—total phenolic content, TFC—total flavonoid content.

**Table 2:** Analysis of variance (ANOVA) and descriptive statistics (R^2^ and CV) of the fitted model.

Term	Regression Coefficient			
Empty Cell	TPC	TFC	DPPH	Rutin
*Intercept*				
β0	1369	1.23	6.39	20.23
*Linear*				
β1	3.15 *	−0.22 *	3.70	7.72 *
β2	0.56	−0.8 *	0.14	3.35 *
β3	3.32 *	0.67 *	0.26	8.643 × 10^−3^
β4	0.42	0.38*	0.065	0.92
*Interaction*				
β12	1.6	0.66 *	0.29	2.59 *
β13	0.88	−0.47	0.11	−5.67 *
β14	−0.34	−0.33	−0.033	1.14
β23	−1.11	−0.12	0.44 *	7.86 *
β24	1.15	0.21	0.10	−0.76
β34	−0.064	0.68 *	0.13	2.64 *
*Quadratic*				
β11	−2.92	−0.094	0.45 *	−1.75
β22	0.48	0.54 *	0.20	−0.89
β33	−1.48	0.41	0.045	5.56 *
β44	0.51	0.027	−0.39 *	−0.36
R2 ^a^	0.8233	0.8393	0.9843	0.6864
CV ^b^	17.22	35.67	6.73	8.5588
pm-value ^c^	0.0034	0.0019	<0.0001	<0.0001
plf-value ^d^	0.2013	0.057	0.0566	0.4860

* *p* < 0.01; ^a^ coefficient of multiple determination; ^b^ coefficient of variance [%]; ^c^ probability of *F* value for the model; ^d^ probability of *F* value for the lack of fit, TPC—total phenolic content, TFC—total flavonoid content.

**Table 3:** Optimized extraction parameters with estimated predicted and observed values.

Optimal Parameters	Predicted Values	Obtained Values
Extraction temperature (°C)	80	80
Ethanol concentration (%)	62.23	62.23
Ultrasound power (%)	56.05	56
TPC (mg GAE/DW)	19.52	19.82
TFC (mg GAE/g DW)	98.64	99.26
DPPH (mg TE/g DW)	21.4475	21.5423
Rutin (mg/g DW)	15.03	15.79

TPC—total phenolic content, TFC—total flavonoid content.

**Table 4:** HPLC analysis of *C. spinosa* extract obtained under optimal conditions.

Compound	Concentration (mg/g)
Gallic Acid	0.0408
Catechin equivalent	2.0675
Caffeic Acid	0.2495
Syringic Acid	0.1927
Sinapic Acid	0.0502
Rutin	15.5051
Quercetin-3-Glucoside	4.2687

## Data Availability

The original contributions presented in this study are included in the article. Further inquiries can be directed to the corresponding author.
